# The Impact of ACOs on Medicare Costs for ADRD Patients in Socially Vulnerable Neighborhoods

**DOI:** 10.1002/alz70860_105631

**Published:** 2025-12-23

**Authors:** Seyeon Jang, Jie Chen

**Affiliations:** ^1^ University of Maryland School of Public Health, College Park, MD, USA; ^2^ The Hospital And Public health interdisciplinary research (HAPPY) Lab, School of Public Health, University of Maryland, College Park, MD, USA

## Abstract

**Background:**

Accountable Care Organizations (ACOs) can improve care coordination and generate savings for individuals with Alzheimer's disease and related dementias (ADRD), particularly in socially disadvantaged neighborhoods. However, residents in these areas are less likely to enroll in ACOs. To assess the cost‐saving potential of ACOs, we examined their effects on Medicare payments for newly diagnosed ADRD patients, focusing on overall neighborhood social vulnerability (SVI) and its subcategories. Additionally, we explored whether continuous enrollment in high‐quality ACOs led to greater cost savings.

**Method:**

We analyzed Medicare data from 2016–2020, integrating the Medicare Beneficiary Summary File, Medicare Shared Savings Program (MSSP) ACO file, Social Vulnerability Index (SVI), and Area Health Resources File. The study included 293,173 Medicare beneficiaries newly diagnosed with ADRD in 2017 who remained in fee‐for‐service throughout the study period. We tracked Medicare payments from the year before diagnosis (2016) to three years after (2018–2020). Neighborhood disadvantage was measured using the 2020 SVI and its four subdomains: racial/ethnic minority status, socioeconomic status, housing/transportation, and household characteristics. Beneficiaries were stratified by neighborhood SVI (low <50th percentile, high ≥50th percentile) and ACO enrollment status. Multivariable generalized estimating equation models were applied, including interaction terms to assess whether the impact of ACO enrollment on Medicare payments varied by SVI.

**Result:**

ACO enrollment significantly reduced total Medicare payments across all SVI subcategories after adjusting for various individual‐ and neighborhood‐level characteristics. The greatest savings occurred among ADRD patients in high‐minority neighborhoods. Among ACO enrollees, those in higher‐quality ACOs had progressively lower total payments. Compared to the lowest quality ACOs (≤25th percentile), beneficiaries in the highest quality ACOs (≥75th percentile) experienced a 7% reduction in total payments (*p* <0.001). Continuous ACO enrollment further decreased expenditures, with the lowest spending observed in the highest quality ACOs.

**Conclusion:**

ACOs can significantly reduce Medicare spending for ADRD patients, particularly in socially vulnerable neighborhoods. Policies should target these populations, implement tailored interventions, and expand ACO enrollment to underserved communities.

